# Human health risk assessment of elevated and variable iron and manganese intake with arsenic-safe groundwater in Jashore, Bangladesh

**DOI:** 10.1038/s41598-020-62187-5

**Published:** 2020-03-23

**Authors:** Gopal Chandra Ghosh, Md. Jahed Hassan Khan, Tapos Kumar Chakraborty, Samina Zaman, A. H. M. Enamul Kabir, Hiroaki Tanaka

**Affiliations:** 1 Department of Environmental Science and Technology, Jashore University of Science and Technology, Jashore, 7408 Bangladesh; 2 Research Center for Environmental Quality Management, Graduate School of Engineering, Kyoto University, Otsu, Japan

**Keywords:** Environmental chemistry, Environmental impact

## Abstract

Groundwater through hand-operated tubewell (a type of water well) tapping is the main source of drinking water in Bangladesh. This study investigated iron and manganese concentration in groundwater across Jashore district–one of the worst arsenic contaminated area in Bangladesh. One working tubewell that had been tested previously for arsenic and marked safe (green) was selected from each unions of the district. Results revealed that approximately 73% and 87% of groundwater samples exceeded the limits for iron and manganese in Bangladesh drinking water, respectively. Additionally, spatial distribution of iron and manganese indicate that only 5% of the total surface area of groundwater is covered by safe level of iron and manganese. Human health risk due to ingestion of iron and manganese through drinking water was evaluated using hazard quotients (HQ) for adults and children. The result of the health risk assessment revealed that the non-carcinogenic health risks due to ingestion of iron (HQ up to 1.446 for adults and 0.590 for children) and manganese (HQ up to 2.459 for adults and 1.004 for children) contaminated groundwater are much higher among adults than children. On the basis of occurrences, spatial distribution and health risk assessment results, the area can be categorized as a high-risk zone for iron and manganese-related problems and needs special attention in order to protect public health of local residents.

## Introduction

Groundwater is the main source of drinking water in Bangladesh and over 90% of the country’s drinking water source is groundwater. Now, it is estimated that close to 98% of the population of Bangladesh have access to ‘technologically improved water source’ but this level is likely lower than 39% when considering Sustainable Development Goal water target (SDG goal 6.1)^1^ of ‘safely managed water’^2^. Safely managed water services ensure access to a technologically improved water source which is available when needed, located on premises and free of fecal and priority chemical contamination.

In Bangladesh, access to technologically improve water source came mainly due to installation of tubewells–a type of water well, a low-cost technology used to withdraw groundwater by hand powered. Since their large scale installation in the 1970s and 1980s for obtaining microbiologically safe water, tubewells have become popular in Bangladesh, with close to 95% of rural people and 70% of urban people using them today^3^. Even though groundwater can be a safe and reliable source of drinking water, it can also be affected by geogenic contaminants, such as arsenic, iron and manganese. Therefore, increased consumption of groundwater in some area has led to long-term exposures to arsenic^4^, iron^5^ and manganese^6,7^ that are associated with adverse health effects.

Iron and manganese are naturally occurring metals, and coexist in groundwater as they have many common chemical properties including similar valence charge in physiological conditions, ionic radius and absorptive mechanisms for individuals^8,9^. Iron and manganese are essential elements required for proper body function. The World Health Organization (WHO) has established secondary or aesthetic based water guideline of iron as 0.3 mg/L, and derived a health-based drinking water guideline value of manganese as 0.4 mg/L^10^. Similarly, for aesthetic, economic, and health-related reasons, Bangladesh has established standards of 0.3 to 1.0 mg/L for iron and 0.1 mg/L for manganese. Manganese is ubiquitous in common foods, and its deficiency is rare in humans. Generally, dietary manganese intake greatly exceeds that from drinking water. In rural Bangladesh, it was speculated that high body iron stores were due to high levels of iron in the groundwater^11^. However, overexposure to these metals can cause adverse health risks including, parkinson disease, huntington disease, cardiovascular disease, hyperkeratosis, diabetes mellitus, pigmentation changes, alzheimer disease, kidney, liver, respiratory and neurological disorders^6,12–14^. According to the National Drinking Water Quality Survey (NDWQS) in 2009^15^, approximately 70% and 61% of drinking water sources in Bangladesh(n = 2896), mainly groundwater, have been found to contain elevated level of iron (>0.3 mg/L) and manganese (>0.1 mg/L), respectively. However, the same survey results revealed that arsenic in only 8% of the samples exceeded the Bangladesh standard (0.05 mg/L)^16^, while 18% exceeded the WHO guideline value (0.01 mg/L)^10^. As a result, tubewell water contain safe level of arsenic may contain elevated level of iron and manganese. The British Geological Survey (BGS) of Groundwater Studies of Arsenic Contamination in Bangladesh— a systematic survey of 61 of the 64 districts of Bangladesh, also identified elevated and variable level of iron and manganese in tubewells water^4^. The survey results revealed that average (minimum, maximum) iron and manganese concentration (mg/L) were 2.81(0.027, 10.5) and 0.38(0.39, 2.6), respectively in Jashore district^4^. According to the NDWQS survey of 2009^15^, 42% and 80% of tubewells (n = 43) in Jashore district contain iron and manganese, respectively, above Bangladesh drinking water standards. In Bangladesh, testing of tubewell water is not mandated by regulations; therefore, tubewell water is not monitored consistently. Since 1993, the first detection of arsenic in groundwater in Bangladesh, the Government with help from international and national organizations, has tried to inform people about the presence of arsenic in drinking water and associated health risk. Several actions have already been taken to provide access to safe (As < 0.05 mg/L) drinking water in arsenic contaminated areas mainly through the installation of tubewells at targeted deeper depth aquifer^2^. However, iron and manganese issues have attracted relatively less attention so far for safe water supply^2^.

The objectives of this study are to investigate the level of iron and manganese in tubewells water which contain safe level of arsenic in Jashore district of Bangladesh, and assess the health risk of the exposure of the local residents to iron and manganese. This study also uses geographic information system (GIS) for the identification of risk areas. Groundwater through tubewell tapping is the main source of drinking and cooking water in the study area. Therefore, this study has great importance for the identification of options for groundwater remedy and management to protect public health.

## Materials and Methods

### Study area, tubewells selection and sample collection

**Figure 1:**
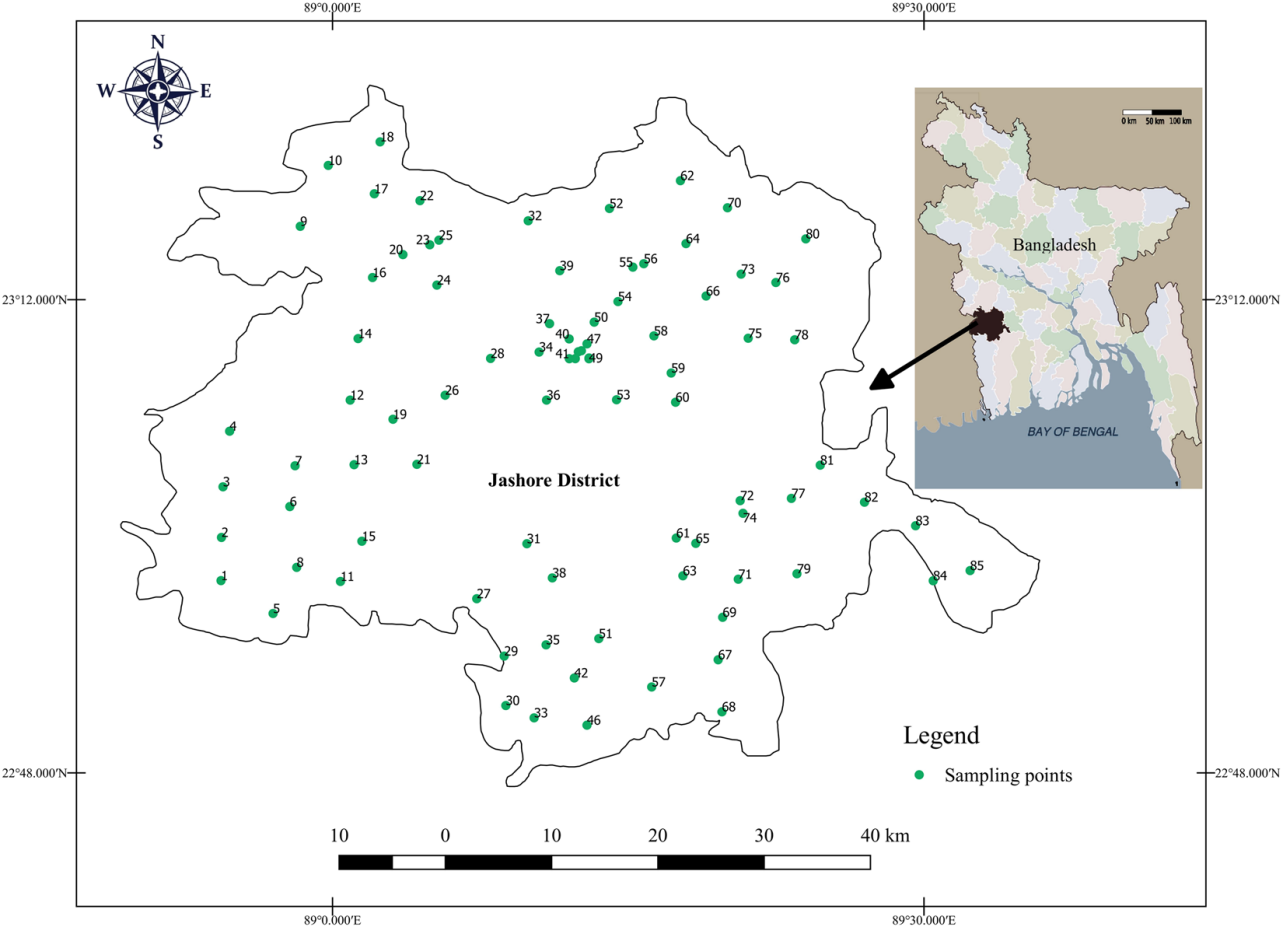
Location map of the study area (Jashore District) and distribution of sampling points.

This study was performed in Jashore district with an area of ~2,606 km^2^ and an estimated population of 2,764,547 located in the rural southwestern part of Bangladesh (Fig. 1). Similar to other area of Bangladesh, groundwater is the main source of drinking water in this district. The arsenic contamination in this district’s groundwater is among the worst in the country. One working tubewell that had been tested previously for arsenic and found safe level of arsenic (As < 0.05 mg/L) was selected for water sampling from each unions (the smallest rural administrative and local government units in Bangladesh) of the district. Some other specific criteria to select the tubewells were: operating full year round, installed in public place, heavily used (more than 20 households depends on it), and mainly used for drinking purposes. Finally, a total of 85 out of 93 unions of the district were selected for water sampling (Fig. 1). A majority of the tubewells (n = 48, 56%) were installed within 10 years of the survey (median (interquartile range, IQR) year of installation: 2008 (2001, 2012)). Median (IQR) depth (feet) was 180 (160, 200). The samples were collected from January through March 2017. Eight unions were excluded in this study due to water logging in those unions during sampling time; the selected tubewells not function and people of those unions used drinking water from nearby unions. Geographical positions were recorded during the sampling by using GPS. Tubewells water samples were collected directly from the tubewell water flow in high density polyethylene (HDPE) bottles (1 L) after 5 or 8 min pumping, depending on depth, in a steady stream of water to reach the aquifer water source. The samples were acidified to pH <2.0 at sampling location using 2 mL of nitric acid (69% pure, ACS reagent grade, Sigma-Aldrich) and transferred to the laboratory for analysis.

### Sample analysis

All the samples for laboratory analysis were filtered through Whatman® glass microfiber filters (Grade GF/B: 1.0 μm) before analysis. Iron and manganese were measured by the FerroVer® (HACH method 8008) and PAN (HACH method 8149) method, respectively, using a HACH DR/3900 Spectrophotometer (HACH, USA).The detection limit of the method is 0.02 to 3.0 mg/L for iron, and 0.01 to 0.7 mg/L for manganese. All glassware used in the experiments was thoroughly cleaned with 1% HNO_3_ (ACS reagent grade, Sigma-Aldrich). Double distilled water was used for standard preparation and sample dilution. Reagent blanks and replicate samples were performed during analysis.

### Statistical analysis and GIS

Statistical analysis, including box and whisker plots, and frequency distribution were performed using R Studio (with R version 3.5.1, www.rstudio.com), and figures were generated using ggplot2 (version 3.1.0). In order to process geographical data and make related calculations, the software QGIS version 2.2.0-Valmiera was used. The inverse distance weighting (IDW) method was chosen for interpolation.

To explore the distribution of iron and manganese concentration in the study area, three scale categories defined as ‘minimal’, ‘elevated’ and ‘high’ were used based on established recommendations for iron and manganese in water and daily dietary intake (Table 1). Tubewells were defined as having ‘minimal’ iron and manganese if they were below the WHO aesthetic limit for iron (0.3 mg/L) and Bangladesh standard for manganese (0.1 mg/L) in drinking water, respectively. Tubewells with iron and manganese concentration above Bangladesh drinking water standard but bellow the Joint FAO/WHO Expert Committee on Food Additives (JECFA) defined limit (2.0 mg/L for iron and 0.4 mg/L for manganese) are defined as ‘elevated’^17,18^. The JECFA limit was established by allocating 10% and 20% of the intake to drinking water for iron and manganese, respectively, defined as 0.8 mg iron/kg and 0.2 mg manganese/kg of body weight assuming average body weight of 60 kg and daily water consumption of 2 L^17,18^. Tubewells were defined ‘high’ levels of iron and manganese if the iron and manganese concentrations were greater than 2.0 mg/L and 0.4 mg/L, respectively.

**Table 1:** Distribution of 85 tubewells manganese and iron concentration categories based on Bangladesh, WHO and JECFA drinking water quality guideline.

	Category	N (%)	Mean	Min	Max	Median	IQR
Manganese (mg/L)	Minimal (0–<0.1^a^)	11 (13%)	0.05	0.02	0.08	0.04	0.03, 0.06
	Elevated (0.1–0.4^b^)	36 (42%)	0.22	0.11	0.36	0.21	0.16, 0.27
	High (>0.4)	38 (45%)	0.93	0.40	2.11	0.78	0.55, 1.07
Iron (mg/L)	Minimal (0–<0.3^c^)	23 (27%)	0.14	0.02	0.29	0.15	0.08, 0.20
	Elevated (0.3–2.0^d^)	38 (45%)	0.75	0.31	1.94	0.65	0.39, 0.98
	High (>2.0)	24 (28%)	3.72	2.10	6.20	3.56	2.97, 4.40

^a^Bangladesh drinking water standard^16,18^; ^b^WHO health-based value^10^; ^c^WHO aesthetic cut-off^10^;^d^JECFA provisional maximum tolerable daily intake for iron in water^17,18^.

### Health risk assessment

In this study, health risk assessment method proposed by United States Environmental Protection Agency (US EPA)^19^ was used and population exposed to iron and manganese contaminated groundwater by ingestion via drinking tubewells water was considered. Iron and manganese are both non-carcinogens according to the division of toxicants, as suggested by US EPA. Non-carcinogenic human health risk associated with ingesting iron or manganese was assessed according to a hazard quotient (HQ)^19^ as Eq. (1):

1 $${\rm{H}}{\rm{Q}}=\frac{{\rm{C}}{\rm{D}}{\rm{I}}}{{\rm{R}}{\rm{f}}{\rm{D}}}$$

where RfD (mg/kg/day) is the reference dose of iron or manganese as suggested by US EPA (0.7 mg/kg/day for iron^20^ and 0.14 mg/kg/day for manganese^18^, respectively). CDI is the chronic daily intake^19^ of iron or manganese (mg/kg/day), and calculated using Eq. (2):

2 $$CDI=\frac{{\rm{C}}\times {\rm{I}}{\rm{R}}\times {\rm{E}}{\rm{F}}\times {\rm{E}}{\rm{D}}}{{\rm{B}}{\rm{W}}\times {\rm{A}}{\rm{T}}}$$

where C is the iron or manganese concentration in groundwater (mg/L); IR is the human water ingestion rate in L/day (3.53 L/day for adults^21^ and 1.0 L/day for children^19^); ED is the exposure duration in years (70 years for adults and 6 years for children^19^); EF is the exposure frequency in days/year (365 days for adults and children); BW is the average body weight in kg (50 kg for adults^22^ and 15 kg for children^19^), and AT is the averaging time (AT = 365 × ED(d)).

Based on the HQ values, no significant health risk of non-carcinogenic effects is anticipated if the value is less than one (HQ < 1). However, in the case of HQ value that exceeds one (HQ > 1), residents are exposed to non-carcinogenic health risk. For the risk assessment of iron and manganese in combination in the drinking water, a hazard index (HI) was employed by summing the calculated HQ values of iron and manganese.

A HI of less than one (HI < 1) means iron and manganese are unlikely to cause adverse non-cancer health effects over a lifetime of exposure. However, HI greater than one (HI > 1) mean adverse non-cancer health effects are likely.

## Results and Discussion

### Iron and manganese in groundwater

**Figure 2:**
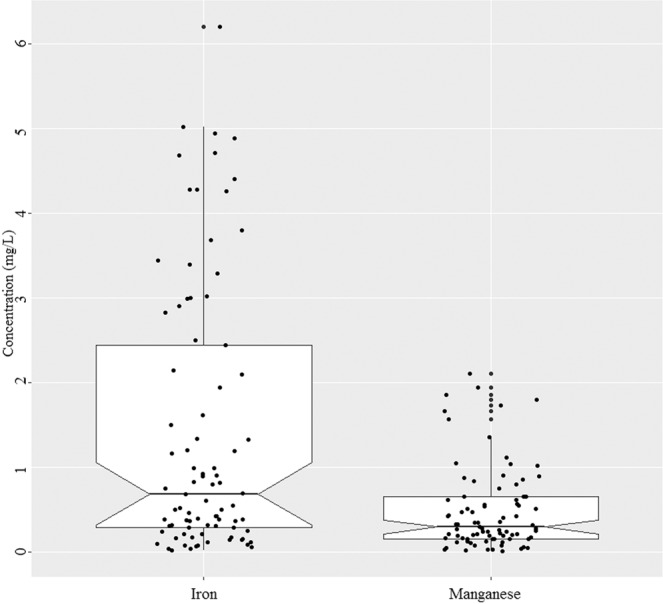
Notched box plot of iron and manganese concentration in the tubewells water (n = 85). The box bounds the interquartile range (IQR) divided by the median and whiskers extend to 1.5 × IQR beyond the box. The notch top and bottom indicate a 95% confidence interval (CI) for the median. Small disks represent the individual data points.

**Figure 3:**
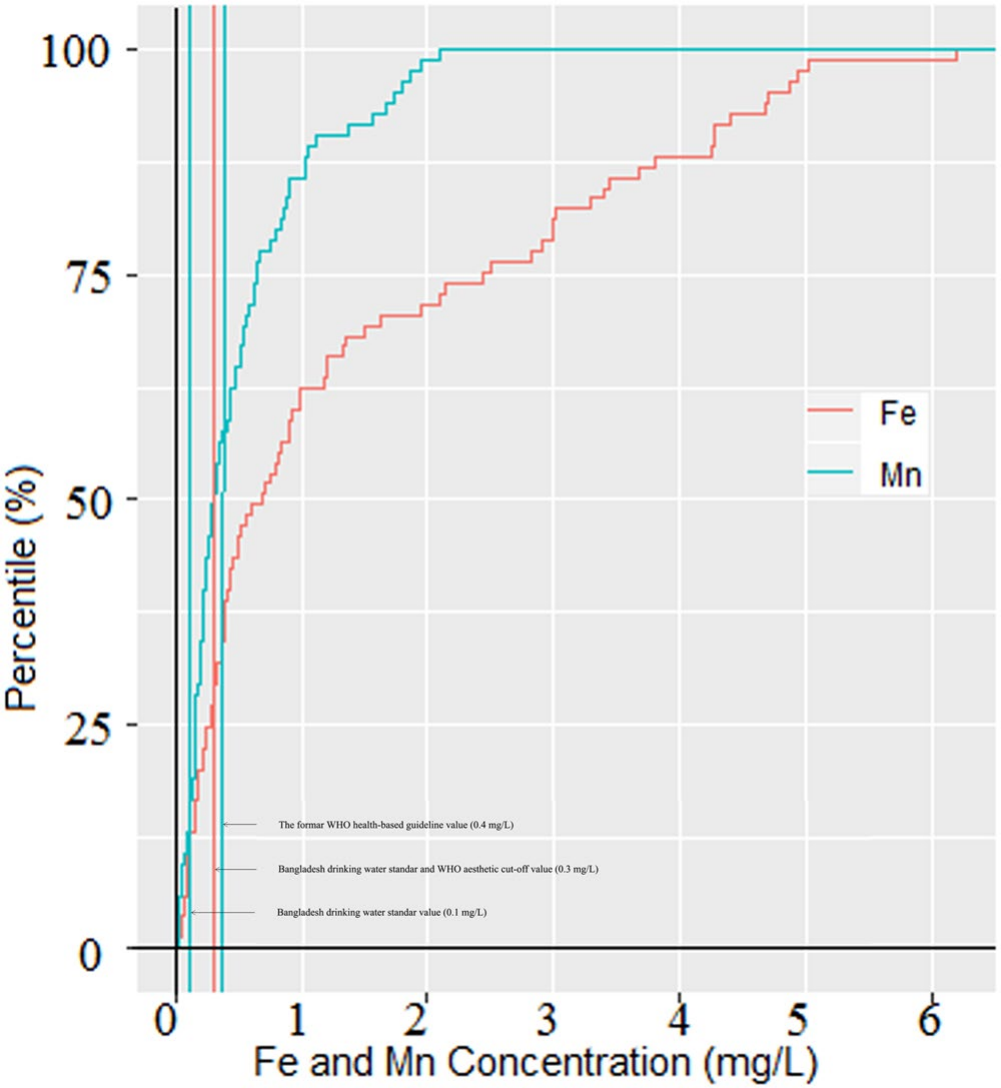
Cumulative frequency plot for tubewells iron and manganese concentration (n = 85).

As mentioned above, this study focused on iron and manganese concentration in 85 tubewells water with safe level of arsenic (As < 0.05 mg/L) across Jashore district (Fig. 1). The tubewells iron concentrations showed a wide range (0.02 to 6.2 mg/L) with a median iron concentration of 0.69 mg/L and inter quartile range 0.27 to 2.47 mg/L (Fig. 2). Approximately 73% of the tubewell water exceeded the limit for iron of which 45% were defined as ‘elevated’ and 24% were defined as ‘high’ —JECFA provisional maximum tolerable daily intake for iron in water (Table 1; Fig. 3). The maximum value of iron and manganese were nearly 20 and 21 times higher than the lower limit of Bangladesh standard of iron (0.3 to 1.0 mg/L) and manganese (0.1 mg/L), respectively. The mean value of iron was 1.4 mg/L, which was 4.6 times that of iron limit. The results are in agreement with elevated and variable levels of iron in tubewells water in Bangladesh^15,17^. Merrill *et al*. (2010)^5^ found iron concentration in a northeastern Bangladesh to be higher (median = 7.6 mg/L, inter quartile range = 1.6 to 17.6 mg/L) than this study. Elevated level of iron (3.9 to 10.5 mg/L) was previously reported from a small village in Jashore district^23^.

**Figure 4:**
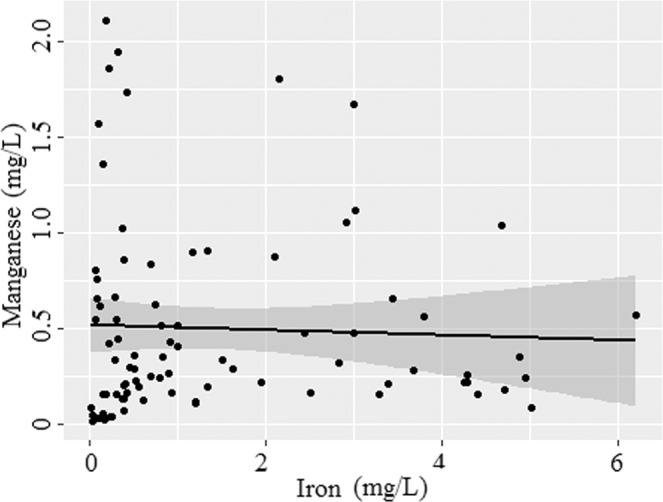
Manganese versus iron concentration in the tubewells water (n = 85). Solid line depicts the predicted relationship between iron and manganese; and shaded regions are the 95% confidence intervals.

The tubewells manganese concentrations also showed a wide range (0.016 to 2.108 mg/L) with a median concentration of 0.298 mg/L and inter quartile range 0.162 to 0.654 mg/L (Fig. 2). The mean value of manganese was 4.7 times higher than the Bangladesh standard of 0.1 mg/L, but remained slightly high to the former WHO health guideline of 0.4 mg/L. Similar to iron, a majority of the tubewells (87%) had manganese concentrations higher than the Bangladesh standard (0.1 mg/L) and only 13% of tubewells had ‘minimal’ (Table 1; Fig. 3). Many tubewells water have low iron levels but high manganese (Fig. 4). Other tubewells show the opposite relation: low manganese and high iron concentrations. However, there were no strong correlation between iron and manganese in the tubewells water (Fig. 4). A nationwide less densely sampled by British Geological Survey are in agreement with this result, where 74% of the wells water manganese concentration exceeded the limit^4^. Elevated levels of manganese were also reported in portions of Araihazar, Bangladesh^24^.

### Spatial distribution of iron and manganese

**Figure 5:**
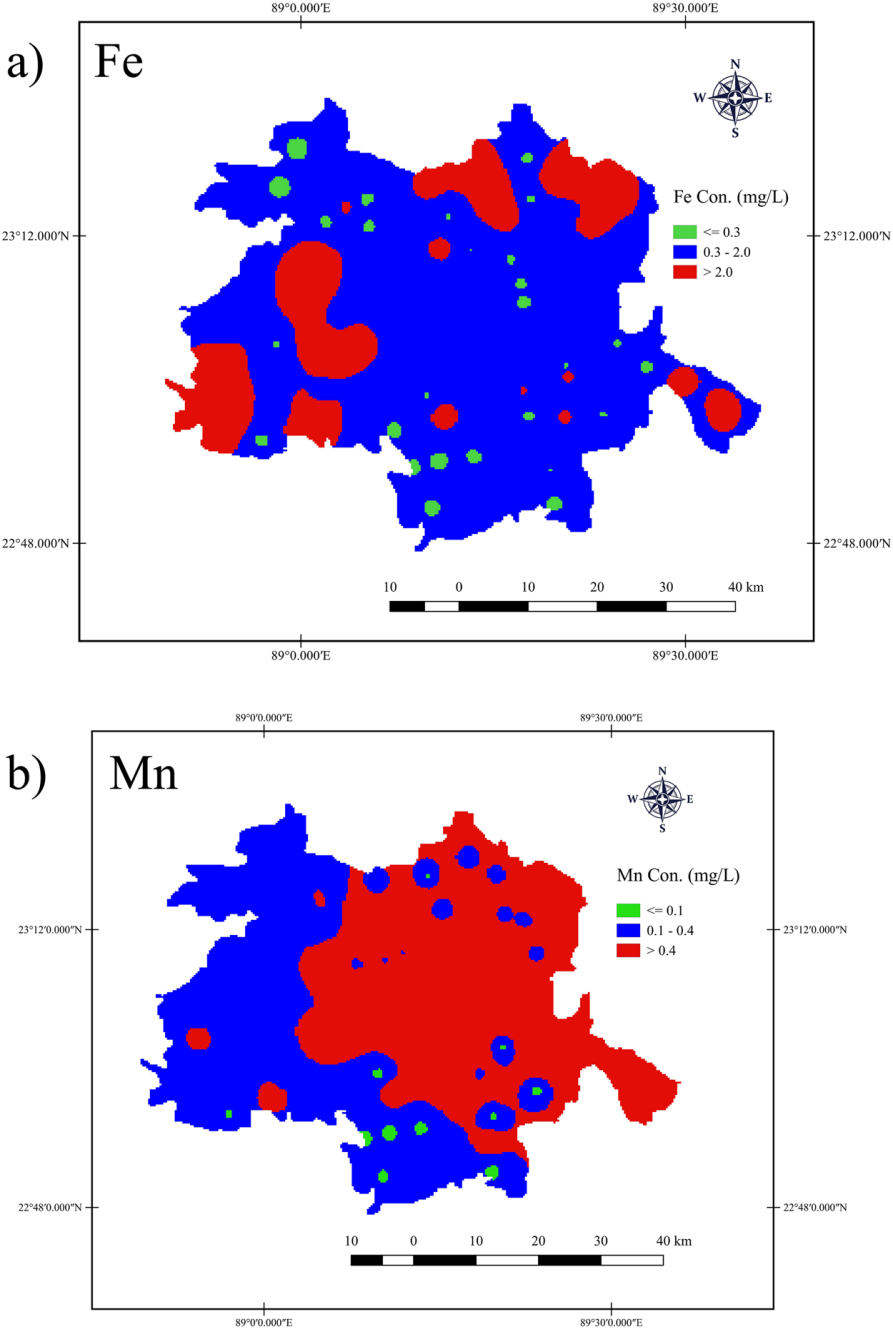
Spatial distribution maps of (**a**) iron and (**b**) manganese in the groundwater of Jashore district.

Figure 5 showed the spatial distribution of iron and manganese in the groundwater of Jashore district based on health related class boundaries. For iron, more than 75% of the total surface area of groundwater were covered by ‘elevated’ level of iron (0.3 to 2.0 mg/L) followed by ‘high’ level of iron (>0.2 mg/L) that covered 20% of the total surface, and only 5% covered by ‘minimal’ level of iron (<0.3 mg/L) (Fig. 5a). The ‘high’ levels of iron are distributed mostly in the southwestern and northeastern part of Jashore district. On the other hand, high level of manganese (>0.4 mg/L) covered 60% of the total surface area of the groundwater and distributed in the central and eastern part of the district (Fig. 5a). The ‘elevated’ level of manganese (0.1 to 0.4 mg/L) is distributed in the western and southern part of Jashore district and covered more than 35% of the total surface area of groundwater, and only less than 5% of the total surface is covered by ‘minimal’ level of manganese (<0.1 mg/L) that are distributed scatteredly in the southern part of the district (Fig. 5b). Moreover, only less than 5% of the total surface area is covered by ‘minimal’ level of both iron and manganese.

### Assessment of human health risks

**Figure 6:**
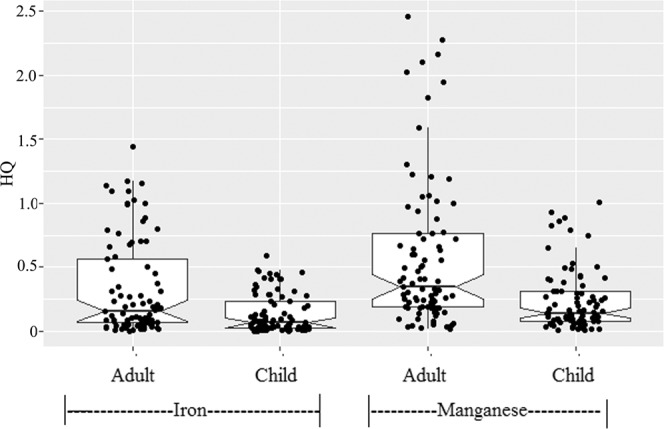
Notched box plot for non-carcinogenic hazard quotient (HQ) of iron and Manganese for different individuals. Boxplot details as in Fig. 2.

In this study, only oral ingestion of iron and manganese through drinking tubewells water was taken into consideration for adults and children health risk assessment. The results of the risk assessment (HQ) are presented in Fig. 6. For adult, the HQ values for iron showed a wide range (0.004 to 1.446) with a median value of 0.161 and inter quartile range of 0.063 and 0.576. As show in Fig. 6, HQ values of iron for adults were higher than 1 in around 8% tubewells (n = 7), which indicated adverse health effect, although iron in 28% of the tubewells exceeded the JECFA provisional maximum tolerable daily intake for iron in water (Table 1). The HQ values of iron for children also showed a wide range (0.002 to 0.590) with lower median value (0.066) and inter quartile range (0.026 to 0.235) than adults.

All HQ values of iron for children were less than 1 (Fig. 6), suggesting no potential non-carcinogenic health concerns among children. Iron in available form in drinking may provide nutritional benefit. Studies have shown that iron in drinking water that is highly bioavailable could be absorbed as high as 40%^25–27^. A study in Bangladesh suggested that a positive association between groundwater iron contents (between above or below 1 mg /L) and linear growth of children with iron deficiency^28^. However, excessive body iron may be an issue of concern because of a possible association with several chronic diseases, such as heart disease^29–31^ and diabetes^32,33^.

**Figure 7:**
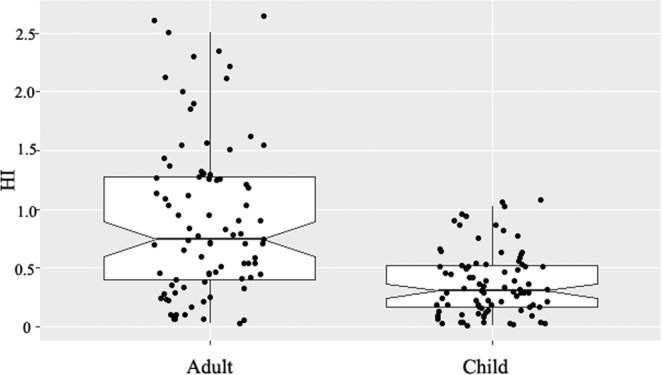
Hazard indexes of different individuals for iron and manganese. Boxplot details as in Fig. 2.

For adults, the HQ values for manganese showed a wide range (0.019 to 2.459) with a median value of 0.348 and inter quartile range of 0.186 and 0.764. As for manganese, the HQ values for children are lower than adults, and the maximum value for children is 1.004. The HQ of manganese for child was more than 1 at only one tubewell but HQ values of manganese for adults were higher than 1 at sixteen tubewells, which indicated relatively high health risk. In the studied tubewells, manganese concentration exceeded the limit (0.1 mg/L) for Bangladesh drinking water in around 83% tubewells, whereas 45% of the tubewells exceeded the WHO guideline value (0.4 mg/L). Wasserman *et al*.^6^ showed a positive association between manganese concentrations higher than 0.4 mg/L and reduced intellectual function of children aged 10 years in Bangladesh. A cross-sectional study in Bangladesh also suggested infants exposed to drinking water manganese concentrations higher than 0.4 mg/L had increased mortality risk during the first year of life when compared with unexposed infants^34^. A recent study also showed a positive association between manganese concentrations in drinking water and reduced intellectual function of children ages 5.9–13.7 years in Canada^7^. As shown in Fig. 7, the HI values for children showed a wide range (0.165 to 1.650) with a median value of 0.407 and inter quartile range of 0.223 and 0.894. HI values for children were found higher than 1 (HI > 1) at three points, indicating possible health hazards in Jashore district with respect to the use of groundwater with elevated level of iron and manganese for children drinking purpose. For adults, around 39% of the tubewells (n = 33) showed HI values higher than 1, which indicate relatively high health threat (Fig. 6).

### Limitations

In this study, tubewells which had been tested previously and marked safe (green; As <0.005 mg/L) was selected for water sampling to measure iron and manganese. We did not measure As concentration. The samples were collected only in dry season and no temporal variability was studied, which is a limitation of this study. Eight water logged unions were excluded and no attempt was made to go back at different times to collect sample as those areas experiencing seasonal water logging. Additionally, bioaccessibility of iron and manganese in groundwater was not measured and thus could not estimate actual iron and manganese exposure. Moreover, applying the risk assessment on these measurements are therefore even more compelling. We expressed non-carcinogenic risk of iron and manganese in terms of a hazard quotient (HQ), and hazard index (HI) for iron in combination with manganese. Elevated and variable level of iron and manganese in groundwater in Jashore, Bangladesh could represent a potential risk, but there is no way to establish that risk with any certainty. This study finding, associated with the absence of reports of iron and manganese deficiency in human, led us to conclude that the possible consequences of excess exposure to iron and manganese from groundwater deserve further attention.

### Conclusions and recommendations

In this study, a total of 85 tubewells water samples were investigated to assess the iron and manganese concentrations in the groundwater of Jashore district, which is located in arsenic-polluted southwestern part of Bangladesh. Approximately 73% and 87% of the samples exceeded Bangladesh limits for iron and manganese in drinking water, respectively. The result of the health risk assessment revealed that the non-carcinogenic health risks due to ingestion of iron and manganese contaminated groundwater are much higher among adults than children. Additionally, other inorganic and organic contaminants in the groundwater may pose a threat to human health. Furthermore, it is necessary to consider the iron and manganese bioavailability in drinking water and in the rest of the human diet to integrate this value into a risk assessment. Therefore, it is important to communicate with people who intake these elements in the region to limit their consumption. Finally, special attention should be paid to reduce iron and manganese concentration in tubewells water with safe level of arsenic, and special measures need to be taken to protect children, the most vulnerable population, from iron and manganese toxicity. To improve this situation, several steps including periodical testing of tubewells water quality, increased awareness, entrepreneurial development and incentive to allow business to supply safe water at low cost, development of low cost household water treatment systems, appropriate water safety planning and strategies at the household level need to be taken to reduce the risk. Further research is needed to acquire a better understanding of the individual health risks associated with iron and manganese in drinking water sources.
